# Evolution of Aortic Root Preservation After Type A Acute Aortic Dissection Repair: Impact of Root-Residual Aortic Dissection

**DOI:** 10.3390/jcm14176274

**Published:** 2025-09-05

**Authors:** Alizee Porto, Axel Court, Pierre Antoine Barral, Mohamed Boucekine, Laura Imbert, Vlad Gariboldi, Alexis Jacquier, Frederic Collart, Alexis Theron, Marine Gaudry

**Affiliations:** 1 Department of Cardiac Surgery, APHM, Timone Hospital, 13005 Marseille, France; axel.court@ap-hm.fr (A.C.); vlad.gariboldi@ap-hm.fr (V.G.); frederic.collart@ap-hm.fr (F.C.); alexis.theron@ap-hm.fr (A.T.); 2 Department of Radiology, APHM, Timone Hospital, 13005 Marseille, France; pierre-antoine.barral@ap-hm.fr (P.A.B.); alexis.jacquier@ap-hm.fr (A.J.); 3 Department of Epidemiology and Public Health Cost, Assistance Publique-Hôpitaux, 13005 Marseille, France; mohamed.boucekine@ap-hm.fr (M.B.); laura.imbert@ap-hm.fr (L.I.); 4 Timone Aortic Center, Department of Vascular Surgery, APHM, Timone Hospital, 13005 Marseille, France; marine.gaudry@ap-hm.fr

**Keywords:** residual aortic dissection, aortic root, type A aortic dissection, aortic regurgitation

## Abstract

**Background:** The management of the aortic root in type A aortic dissection (TAAD) is challenging. Conservative aortic root repair is considered a reasonable option in most cases. This study aimed to analyze medium-term mortality and aortic root reintervention after TAAD. **Methods:** All patients with TAAD underwent prospective follow-ups between 2017 and 2021. The primary endpoint was the increase in aortic root diameter and the rate of proximal root reintervention. The secondary endpoints were the risk factor and location for root-RAD, and risk factors for severe and moderate AR and mortality during follow-up. **Results:** A total of 210 patients were included, of which 166 (79.0%) did not have root-RAD and 44 (21.0%) had root-RAD after supracoronary aortic replacement. After a mean follow-up of 41.1 months (0–60), four patients (1.8%) died. No cases of aortic root rupture were reported. Two patients in the root-RAD group underwent aortic root reoperation. The aortic root diameter increased by 0.5 mm/year and 0.09 mm/year in the root-RAD and no-root-RAD groups, respectively (*p* = 0.18). Root-RAD was a risk factor for severe AR (HR 19.6; 95%CI: 1.6–2.7; *p* = 0.05) and an independent risk factor for moderate AR (HR 23.0; 95%CI: 2.8–2.9; *p* = 0.009). **Conclusions:** Without increasing long-term mortality, root-RAD increases the risks of AR and subsequent aortic root reintervention. Long-term follow-up is essential for monitoring aortic valve function.

## 1. Introduction

Acute type A aortic dissection (TAAD) is an uncommon disease [1] with a poor short-term prognosis if emergency surgery is not performed [2,3,4]. Surgery involving open repair and replacement of the ascending aorta is the gold standard, as it improves survival and is associated with a 30-day mortality rate ranging from 10% to 20% at expert centers [5].

The management of the aortic root is difficult for surgeons in these emergency situations. Aortic root replacement is used in 10–40% of patients [6,7,8,9,10,11] and is generally performed in cases of existing aortic root aneurysms, extensive destruction of the sinuses or coronary ostia, or connective tissue disease. Surgical options include complete root replacement using the Bentall procedure or valve-sparing root repair using the Yacoub or David procedure. This method of root management can reduce the risk of future aortic root reoperation [7,9,12]; however, it can be considered an aggressive technique owing to its increased risk of perioperative mortality [6,13,14].

To reduce the complexity of proximal procedures, conservative aortic root repair is considered a reasonable option in most cases [15]. Various surgical strategies associated with supracoronary aortic replacement (SCAR), such as root repair using the sandwich technique with Teflon inlay patches [10] and the aortic valve resuspension technique [16,17], have been used. These conservative techniques can be performed quickly and easily, especially for patients with severe clinical conditions, with lower short-term mortality than the root replacement approach [10].

Freedom from proximal aortic reintervention is estimated to be between 75% and 90% at 10 years [9,18,19]. The indication for proximal reintervention was the development of an aortic root aneurysm (aortic root dilatation, false aneurysm) associated with severe aortic regurgitation (AR). The risk factors for the development of an aortic root aneurysm after surgery for TAAD, including root-residual aortic dissection (root-RAD), are not clearly defined.

Regarding these data, this study aimed to analyze medium-term mortality and reintervention of the aortic root after performing ascending aortic replacement on TAAD patients.

## 2. Materials and Methods

### 2.1. Study Population

In this prospective single-center study, all patients who underwent TAAD between January 2017 and December 2021 were included. Patients who underwent aortic root replacement surgery, patients without postoperative computed tomography (CT) scans (not alive after intensive care unit), patients who died in the hospital, and those who declined to participate in follow-up were excluded. Preoperative and intraoperative demographic data were collected. Redo cardiac surgery was defined as cardiac surgery in patients with a history of previous cardiac surgery by sternotomy.

Written informed consent was obtained from each patient after surgery and before discharge from the hospital. All patients included in this study were informed that their data would be used for clinical research. Follow-up was carried out at our Aortic Center, which verified that informed consent had been obtained. The institutional review board approved the project (approval number 2019-48).

### 2.2. Surgical Procedure

The initial surgery for TAAD was performed using cardiopulmonary bypass (CPB), the aortic cross-clamp (ACC), circulatory arrest (CA), and retrograde cold blood cardioplegia. Where possible, moderate hypothermia (28–32 °C) and anterograde cerebral perfusion were achieved with right axillary or carotid artery cannulation. Otherwise, deep hypothermia (18 °C) was achieved by femoral cannulation. Replacement of the ascending aorta, hemiarch aorta, partial arch replacement, or total arch replacement was performed depending on the location of the primary entry tear, the extent of the dissection, and the patient’s condition.

Whether to replace the aortic root depends on whether there are tears in the sinuses, whether there is extensive dissection coronary ostia, and whether there is significant dilatation of the root [20].

In patients with normal aortic root diameter (diameter < 40 mm) with no intimal tear in the aortic root, SCAR was realized. In patients with an aortic root diameter >50 mm or dissections with coronary ostia involvement, aortic root replacement was performed. In patients with moderate aortic root involvement (an aortic root diameter of 40–50 mm) or with dissection of the aortic sinus, the decision to conserve or replace the aortic root was made on a case-by-case basis, as in other surgical centers [21,22,23,24]. An external Teflon-pledged reinforcement or a sandwich technique (with external and internal Teflon-pledged reinforcement) was used.

### 2.3. Endpoints

The primary endpoint was the combined endpoint, which included the increase in aortic root diameter and the rate of proximal root reintervention. The secondary endpoints were the risk factor and location for root-RAD, the rate and risk factors for severe and moderate AR, and all-cause mortality during the follow-up period.

### 2.4. Root-RAD Diagnostic and Follow-Up CT Protocol

All patients underwent a postoperative electrocardiogram-synchronized CT scan, before being discharged from hospital, which was designated as the reference CT scan for diagnosing root-RAD. Root-RAD was diagnosed based on the presence of an intimal flap at the aortic root witch true and false lumen, as confirmed by a senior radiologist during analysis.

All patients who followed up at our aortic center were included in the prospective follow-up. Follow-up was systematically carried out with a surgical consultation and a CT scan at 3, 6, and 12 months, as well as annually in cases of favorable progress. In case of evolution of the aortic root (a +2 mm increase in aortic diameter compared to the previous scan), monitoring was carried out more closely.

Three-dimensional imaging software (OSIRIX software, version 12.5.2, Geneva, Switzerland) was used for image analyses and measurements. Multiplane reconstructions were used to assess the dimensions of the aortic root perpendicular to the axis of blood flow. The standard diameter of the aortic root was measured sinus-to-sinus.

### 2.5. Follow-Up Transthoracic Echocardiography

All patients were examined using transthoracic echocardiography (TTE) by a senior cardiologist postoperatively. During follow-up, TTE was obtained from the patients’ cardiologists. In cases of root-RAD, a TTE was routinely performed at our center to analyze the AR, at each follow-up consultation. The degree of AR was assessed semi-quantitatively or quantitatively and classified as mild, moderate, or severe [25]. Transesophageal echocardiography was performed if necessary.

### 2.6. Aortic Root Reintervention

Indications for reinterventions were an aortic diameter >55 mm, rapid aortic growth (10 mm/year), severe AR in symptomatic patients, or damage to the left ventricle (according to ESC 2021 recommendation) [25]. A multidisciplinary decision was made to validate the indication and surgical technique, depending on the patient’s general condition.

### 2.7. Statistical Analysis

Means and ranges or standard deviations were used to describe continuous variables; categorical variables were described as numbers and frequencies. For categorical variables, the relationships between variables were studied using chi-square test or Fisher’s exact test, as appropriate. For continuous variables, the Wilcoxon test was utilized. The Mann–Whitney U test was used for continuous variables. The normality of the distribution of the variables was assessed using the Shapiro–Wilk test. Logistic regression was used to analyze risk factors for root-RAD, including baseline patient characteristics and surgical technique. Univariable and multivariable models were built to estimate odds ratios (ORs) with 95% confidence intervals (CIs). The survival rate was estimated using the Kaplan–Meier method. The annual rates of root diameter increase were calculated after adjustment for the baseline postoperative diameter of the aortic root. Risk factors for severe AR and moderate AR were analyzed using logistic regression according to the methods of McFadden and Nagelkerke. Univariable models were built to estimate hazard ratios (HRs) with 95% confidence intervals. Multivariable analysis was then performed to estimate adjusted HRs with their 95% confidence intervals. All variables whose *p* value was <0.20 in univariable analysis were considered candidates for the multivariable model. All the statistical tests were two-sided, and a *p* value < 0.05 was considered to indicate statistical significance. Statistical analyses were performed using R-Studio version 2023.03.0 (R-Studio, Boston, MA, USA).

## 3. Results

Between January 2017 and December 2021, 318 patients were treated for TAAD. The in-hospital mortality rate was 12.9%, and the loss to follow-up rate was 6.6% (21 patients). In total, 297 patients were included in our prospective follow-up. Aortic root replacement was performed in 57 patients (19.2%): 53 using the Bentall technique and four using the Tirone David technique. Among these patients, 19.3% (11 patients) died in the hospital.

SCAR was performed in 240 patients (80.8%). The in-hospital mortality rate was 12.5% (30 patients): 18 patients died before postoperative CT, and 12 patients died after postoperative CT (five from stroke, three from multivisceral dysfunction, one from tamponade, one from mesenteric ischemia, one from septic shock, and one from rupture of the descending aorta). Among these 12 patients, none presented with root-RAD.

**Figure 1:**
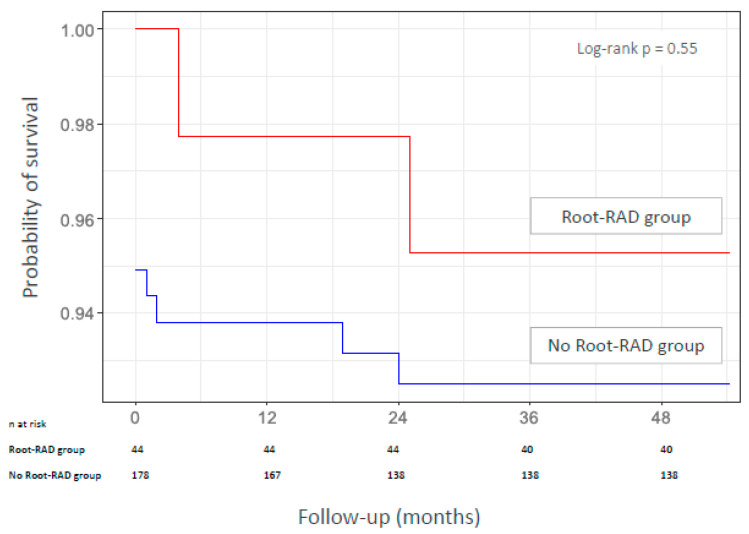
Kaplan-Meier estimate of survival for the root-RAD group and the no-root-RAD group.

In total, 210 patients with TAAD involving the conservation of the aortic root were included: 166 (79.0%) presented with no root-RAD, and 44 (21.0%) presented with root-RAD after surgery before being discharged from hospital (Figure 1).

### 3.1. Baseline and Procedure Data

The mean age was 65.8 ± 11.0 years, 68.6% (144) were male, and 3.3% (seven) had Marfan syndrome or related syndromes. Hemiarch replacement was performed in 153 patients (72.9%). Axillary cannulation patients formed the majority (167 patients, 79.5%). The demographic and procedural data are presented in Table 1.

Concerning the surgical technique used for aortic root preservation, 143 patients (68.1%) underwent external Teflon reinforcement, and 67 patients (31.9%) underwent the sandwich technique: root-RAD was present in 30 patients (21.0%) and 14 patients (20.9%), respectively (*p* = 1.0).

### 3.2. Risk Factors for Root-RAD

During follow-up, no patients in the no-root-RAD group developed root-RAD.

According to univariable analysis, the initial presence of limb ischemia was a risk factor for root-RAD [OD 5.6, 95% CI: 1.2–33.9; *p* = 0.03]. In multivariate analysis, no independent risk factors were identified.

### 3.3. Mortality During Follow-Up

**Figure 2:**
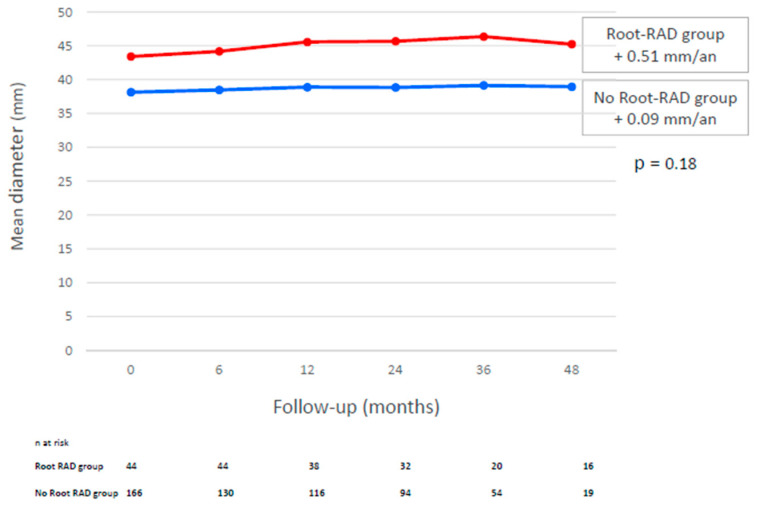
Evolution of vasalva sinus diameter depending on the presence of root-RAD. Comparaison of annual variations in millimeters.

After a mean follow-up of 41.1 months (range 0–60), four patients (1.8%) died during follow-up: two from COVID-19 infection, one from inhalation pneumonitis and one from cancer. The Kaplan–Meier estimated survival rates at 1 year and 5 years were 97.7% [93.4–100.0] and 93.9% [90.3–97.4] and 95.3% [89.1–100.0] and 92.5% [88.6–96.5] in the root-RAD group and the no-root-RAD group, respectively (*p* = 0.55) (Figure 2). No cases of aortic root rupture were reported during the follow-up period.

### 3.4. Increase in Root Diameter During Follow-Up

**Figure 3:**
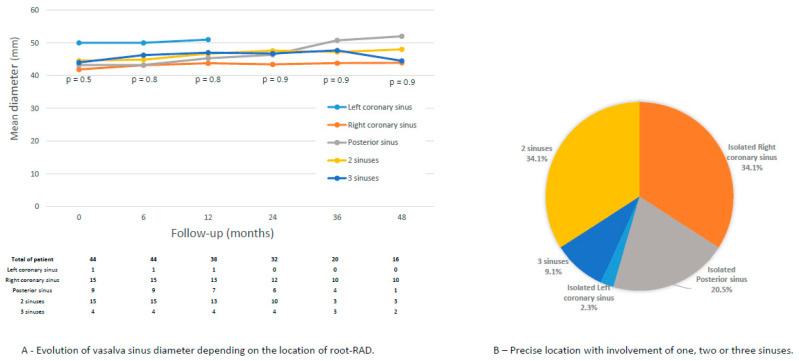
Location of residual dissection in aortic root among 44 patients.

At discharge, the aortic root diameter was 39.2 ± 4.9 mm and 38.2 ± 4.3 mm in the root-RAD and no-root-RAD groups, respectively (*p* < 0.001). During follow-up, these diameters increased by 0.5 mm/year and 0.09 mm/year in the root-RAD and no-root-RAD groups, respectively (*p* = 0.18) (Figure 3). Root-RAD involved the right coronary sinus in 75.0%, the posterior sinus in 61.4%, and the left coronary sinus in 15.9% (Figure 3).

### 3.5. Aortic Root Reintervention

During follow-up, two patients (0.9%) presented with severe AR: two patients (4.5%) in the root-RAD group and zero patients in the no-root-RAD group (*p* = 0.04). Both patients underwent reintervention without postoperative complications (Table 2).

### 3.6. Aortic Regurgitation and Risk Factors

At discharge, 14 patients (6.6%) presented with moderate AR: six patients (13.6%) in the root-RAD group and eight patients (4.5%) in the no-root-RAD group (*p* = 0.04); no patients presented with severe AR. During follow-up, all moderate AR in the no-root-RAD group was reduced and evolved into mild AR. Ten patients (4.7%) presented with moderate AR: 10 patients (22.7%) in the root-RAD group and zero patients in the no-root-RAD group (*p* < 0.001).

A univariable analysis revealed that the presence of root-RAD (HR 19.6; 95% CI: 1.6–2.7; *p* = 0.05), root-RAD involving the right coronary sinus (HR 28.2; 95% CI: 2.2–3.9; *p* = 0.03), and the mean diameter of the aortic root at discharge (HR 1.3; 95% CI: 1.0–1.8; *p* = 0.02) were risk factors for severe AR. According to multivariate analysis, the mean maximal diameter of the aortic root (HR 1.5; 95% CI: 1.0–2.5; *p* = 0.018) was an independent risk factor for severe AR.

According to a multivariate analysis, the presence of root-RAD (HR 23.0; 95% CI: 2.8–2.9; *p* = 0.009) and the mean diameter of the aortic root at discharge (HR 1.4; 95% CI: 1.1–2.1; *p* = 0.02) were independent risk factors for moderate AR (Table 3).

## 4. Discussion

The main findings of the present study are as follows: (i) Replacement of the ascending aorta and preservation of the aortic root in patients with TAAD was the most common technique (80.8%). (ii) One in five patients presented with root-RAD. (iii) The rate of proximal reintervention was low (0.9%). (iv) Reintervention was performed in cases of severe AR influenced by the presence of root-RAD and a large proximal aortic root diameter. (v) The aortic root growth rate was low even with root-RAD.

Reintervention after TAAD is difficult from a technical perspective, and identifying its risk factors is a matter of concern.

In the present study, the rate of proximal aortic reoperation was 0.9% after a mean follow-up of 41 months. Few studies have confirmed this finding. the Nordic Consortium for Acute Type A Aortic Dissection (NORCAAD) Investigators [26] compared the rates of proximal reoperation between supra-aortic root replacement and aortic root replacement in retrospective studies. They reported results that were comparable to those of our study: 73.7% of patients underwent SCAR surgery, 97.8% of patients did not require proximal reoperation 5 years after the initial procedure, and no difference was found between the groups. Similarly, Westby et al. [27] reported two reoperations for aortic valve regurgitation, one of which was due to aortic dilatation, in a series of 89 patients who underwent ascending aorta replacement surgery for TAAD.

Risk factors for proximal reoperation were described by Vendramin et al. [24]: with multivariable analysis, the aortic root diameter at discharge > 45 mm and age were independently associated with proximal reoperation. In our study, the mean aortic root diameter at discharge was a risk factor for AR in patients who were potentially at risk of reoperation. In cases of dilation between 40 and 50 mm in the context of dissection, systematic replacement of the aortic root could be considered. However, this approach is still the subject of debate, and the choice of surgical technique remains a preoperative decision in terms of early survival, long-term durability, and the risk of reoperation. [7,21,22,23,28,29].

Indeed, concerning the choice of root conservation or replacement in cases of TAAD, several studies have compared in-hospital mortality and long-term morbidity rates. No difference was found between different proximal aortic operations: aortic root repair or SCAR [8,13,18,19,30]. Castrovinci et al. [9] reported in a study comparing root replacement and root conservation in TAAD that there was no association between the management of the aortic root and the risk of proximal aortic reoperation (rate of 2.5%). According to the International Registry of Acute Dissection Investigators [7], 35% of patients with TAAD underwent aortic root replacement surgery, whereas 65% underwent ascending aortic replacement surgery. The 3-year mortality rate was similar between these two groups (28.8% vs. 26.4%), as was the proximal aortic reoperation rate (0.8% vs. 0.7%; *p* = 0.770). The authors concluded that using the aortic root surgical technique in emergency patients presenting with acute aortic dissection remains a subject of debate. Recent studies revealed that the incidence of proximal reoperation ranged from 3.0% to 9.0% at 5 years and from 8.0% to 23.2% at 10 years [8,9,15,28,31]. In the present study, the follow-up time was short, which could explain the difference.

In our study, we analyzed the risk factors of root-RAD at discharge, which has not been described in the literature. No preoperative factors were identified as risk factors. In patients with complicated dissection, particularly those experiencing ischemia, the patient’s condition may be critical. In such cases, the surgeon may choose to perform the shortest possible procedure to minimize intraoperative risk. Aortic root replacement may therefore not be performed in this critical context. This could explain why the presence of limb ischemia was a risk factor for root-RAD.

To our knowledge, the present study is the first to analyze the incidence of root-RAD and the impact of conditions on root reintervention. We found that root-RAD occurred frequently (21.0%): 4.5% of these patients developed severe AR and underwent aortic root reoperation, and 22.7% developed moderate AR during the follow-up period.

Aortic regurgitation frequently complicates TAAD in the initial phase and is present in 41% to 76% of patients [32,33]. Various mechanisms of aortic regurgitation have been identified, and aortic valve replacement is not systematic. Indeed, the supracoronary aortic valve resuspension technique restores the geometry of the aortic root and maintains valve competence. Our study found that moderate AR at discharge can be involved in patients with no root-RAD, and aortic root remodeling may explain this result.

In a multivariate analysis, Pessotto et al. [34] reported that preoperative severe AR was a significant risk factor for the development of postoperative aortic valve regurgitation. Kirsch et al. [35] reported that only preoperative severe AR was a significant and independent risk factor for reoperation on the proximal aorta. In our study, the rate of moderate or severe AR during the follow-up period was similar to that reported in other studies [36,37]. We reported that the presence of root-RAD, the location of root-RAD on the right coronary sinus and the mean diameter of the aortic root were risk factors for the development of moderate and severe AR during follow-up. However, moderate AR does not indicate reintervention and long-term follow-up is needed to monitor the outcomes of these patients. Similar to the follow-up of distal RAD [38], long-term follow-up of root-RAD is essential.

Finally, we performed an anatomical analysis and revealed that the annual increase in the Valsalva sinus diameter was on the inframillimeter scale. Residual dissection of the aortic root and its location does not influence these increases in diameter. The baseline Valsalva diameter was 39.2 mm. Jormalaien et al. [11] reported that the diameter of the aortic root increased by 0.23 mm each year after ascending aorta replacement surgery. Ikeno et al. [15] estimated an increase in the diameter of the Valsalva sinus of 0.65 mm/year for a baseline aortic root diameter of 40.2 mm.

## 5. Limitations

Our study has several limitations. This was a monocentric study, which could limit the external validity of this study. We excluded patients without postoperative CT scans (those who were not alive after admission to the intensive care unit), which may have excluded aortic root events. In this study, we did not compare the aortic root preservation technique with the aortic root replacement technique for TAAD. A prospective study comparing the different techniques may be necessary. A small number of root-RAD procedures, a low reoperation rate, and severe AR can also lead to a loss of performance.

## 6. Conclusions

Supracoronary aortic replacement with a conservative aortic root for TAAD is the most common and safest surgery. Without increasing medium-term mortality, root-RAD in patients with large aortic root diameters increases the risk of aortic regurgitation and subsequent aortic root reintervention. Long-term follow-up is essential for monitoring aortic valve function.

## Figures and Tables

**Table 1:** Baseline characteristics and procedure data of the patients. Comparison according to the presence of root-RAD.

	Overall n = 210 (100%)	No Root-RAD n = 166 (79.0%)	Root-RAD n = 44 (21.0%)	*p* Value
**Male sex, n (%)**	148 (66.7)	120 (67.4)	29 (65.9)	0.46
**Age (years), mean (SD)**	65.8 (11.0)	65.3 (11.7)	67.7 (11.0)	0.20
**Hypertension, n (%)**	140 (66.7)	113 (68.1)	27 (61.4)	0.47
**Dyslipidemia, n (%)**	40 (19.0)	33 (19.9)	7 (15.9)	0.67
**Smoking, n (%)**	47 (22.4)	38 (22.9)	9 (20.5)	0.84
**Diabetes, n (%)**	6 (2.9)	5 (3.0)	1 (2.3)	1.00
**COPD, n (%)**	13 (6.2)	8 (4.8)	5 (11.4)	0.15
**CAD, n (%)**	17 (8.1)	12 (7.2)	5 (11.4)	0.36
**Peripheral vascular disease, n (%)**	7 (3.3)	4 (2.4)	3 (6.8)	0.16
**Renal failure, n (%)**	1 (0.5)	1 (0.6)	0 (0)	1.00
**Marfan syndrome, n (%)**	7 (3.3)	4 (2.2)	3 (6.8)	0.16
**Bicuspid aortic valve, n (%)**	5 (2.4)	4 (2.4)	1 (2.3)	1.00
**Redo cardiac surgery, n (%)**	6 (2.9)	5 (3.0)	1 (2.3)	1.00
**Concomitant procedure, n (%)**				
** Innominate artery debranching**	43 (20.5)	39 (23.5)	4 (9.1)	0.04
** Aortic valve replacement**	7 (3.3)	4 (2.4)	3 (6.8)	0.16
**Mean CPB time, min**	148.8 ± 42.5	151.4 ± 43.6	142.0 ± 42.5	0.27
**Mean ACC time, min**	83.6 ± 33.3	85.6 ± 33.4	78.0 ± 33.3	0.08
**Mean CA time, min**	24.4 ± 11.4	25.2 ± 11.2	21.9 ± 11.4	0.20

ACC: aortic cross-clamp; CA: circulatory arrest; CAD: coronary artery disease; COPD: chronic obstructive pulmonary disease; CPB: cardiopulmonary bypass; SD: standard deviation.

**Table 2:** Aortic root reintervention: characteristics of the two patients.

	Age, Years	Location of Root-RAD	Delay of Reintervention	Indication of Reintervention	Reintervention	Follow-Up
**Patient 1**	79	Isolated right coronary sinus	18 months	Severe AR, aortic root 61 mm	Modified Bentall procedure	No postoperative complication. Alive at 2 years.
**Patient 2**	68	2 sinuses (right and posterior)	4 years	Severe AR, aortic root 50 mm, aneurysm in the descending aorta	Hybrid treatment: SCAR with aortic valve resuspension, supra-aortic trunk reimplantation, TEVAR	No postoperative complication. Alive at 1 year.

AR: aortic regurgitation; RAD: residual aortic dissection; SCAR: supracoronary aortic replacement.

**Table 3:** Details of variables in the univariable and multivariable analyses of risk factors for severe or moderate AR during follow-up.

	Univariable Analysis		Multivariable Analysis	
Variables	HR (95% CI)	* p * Value	HR (95% CI)	* p * Value
**Risk of severe AR**				
Root-RAD	19.6 (1.6–2.7)	**0.05**	4.48 (0.2–7.3)	0.27
Location of root-RAD: right coronary sinus	28.2 (2.2–3.9)	**0.03**	4.48 (0.2–7.3)	0.27
Mean diameter of aortic root at discharge	1.3 (1.0–1.8)	**0.02**	0.8 (0.4–1.4)	0.21
Maximum diameter of aortic root	1.4 (1.1–2.0)	**0.006**	1.48 (1.0–2.5)	**0.018**
**Risk of moderate AR**				
Chronic obstructive pulmonary disease	14.0 (3.4–55.1)	**<0.001**	5.5 (0.7–66.5)	0.09
Marfan syndrome	10.5 (1.7–51.6)	**0.005**	4.1 (0.2–2.9)	0.31
Root-RAD	10.1 (12.5–13.1)	**0.002**	23.0 (2.8–2.9)	**0.009**
Mean diameter of aortic root at discharge	1.4 (1.2–1.7)	**<0.001**	1.4 (1.1–2.1)	**0.02**
Maximum diameter of aortic root	1.2 (1.1–1.4)	**<0.001**	0.9 (0.7–1.1)	0.13

AR: aortic regurgitation; CI: confidence interval; HR: hazard ratio; RAD: residual aortic dissection.

## Data Availability

The data underlying this article cannot be shared publicly for reasons of data protection.

## Abbreviations

ACC	Aortic cross-clamp
AR	Aortic regurgitation
AV	Aortic valve
CA	Circulatory arrest
CABG	Coronary artery bypass graft
CPB	Cardiopulmonary bypass
CI	Confidence interval
CT	Computed tomography
Root-RAD	Root residual aortic dissection
SCAR	Supracoronary aortic replacement
TAAD	Type A aortic dissection
TEVAR	Total endovascular aortic repair
